# Colds

**Published:** 1885-06-20

**Authors:** George G. Groff

**Affiliations:** Lewisburg, Penn.


### "COLDS."

BY GEORGE G. GROFF, M.D., LEWISBURG, PENN.

Colds are so common that almost every one has frequently suffered from them, and yet very few persons have an intelligent idea of what a cold is, or that they are ever dangerous, or that they need any treatment at all. They are, to say the least, often very troublesome and annoying, and in some cases do certainly lead to more serious disorders. To know "how to cut a cold short" in a day or two would, to many people, be worth a great deal.

### WHAT IS A COLD?

This question is much more easily asked than answered. At the present time the phenomena of a cold are ascribed to an *inflammatory state* of the system. The body is slightly below par; but an *inflammatory state* sounds strangely where the whole body seems chilled; and, yet, experiment has demonstrated that, when the body is apparently coldest and the patient crouches over the fire, the body heat is one to several degrees *above* the normal. Every one has noticed that, at times, it is very easy to "catch" a cold, while at other times a person may go for many months without one. The reason is, at times the body is in *perfect health*, or nearly so; at other times it is run down, *below par*, and a little exposure or indiscretion develops the latent inflammation, and a cold is "caught." We know that a cold is accompanied by inflammation, for when it has settled in the head or on the chest, the inflammation is very evident. After *free perspiration* the fever at once diminishes, and the body returns to its normal temperature.

### SEAT OF A COLD.

It is often held that a cold has some definite seat in the body. It is true, certain parts are frequently more affected than others. Thus we have "a cold in the head," when the membranes which line the air passages of the head are affected; "a cold on the chest" or lungs, when the organs of the chest seem inflamed and congested ; or the cold may settle in the bowels, and a slight diarrhœa results, or more serious inflammation of the bowels; or the kidneys and the bladder may be the parts most affected; finally, we hear of colds affecting the whole body, including even the bones. The truth is that, in all probability, a number of minor ailments pass under the name of a "cold," which is thus only a means of expressing an unknown complaint.

### HOW TO AVOID COLDS.

Colds often lead to more serious troubles, and all desire to avoid them, if possible. They belong peculiarly to the cold and changeable weather of our autumns, winters and springs, but may be contracted at any season of the year. The following hints will, we think, if followed, enable any one to live for years without a "cold :"

Dress the body warmly, and throughout all cold and changeable weather dress the whole body in warm flannel. Many a heavy doctor's bill may be saved by wearing good red flannel.

Keep the pores of the skin well open by frequent bathing. Some affirm that a cold cannot be caught if the pores are constantly freely open. Bathe in a warm room, if possible, and so arrange it that you do not become chilled while bathing. Bring the body to a glow by a rough towel, after leaving the bath.

Breathe through the nose. This is an important matter. Children should early be taught to so breathe. It is a means of avoiding many diseases.

Avoid draughts of air. Never stand still on a cold, windy day, especially after walking or other exercise; and always avoid standing on ice or snow, or damp ground, or in a cold wind. After exercising always cool off slowly. On a cold day always put on your coat and keep moving while cooling off.

Don't live in a close, stuffy, overheated room. Breathe pure air day and night. Ventilate thoroughly your bed chamber. Those who live in the open air seldom have a cold, while those indoors suffer constantly.

Keep the back, especially between the shoulder blades, well covered and warm. The clothing over the back should be as heavy, if not heavier than over the chest, for the vital organs of the chest are here much exposed. Never lean the back against a cold wall or post.

When about to make a journey, put on extra clothing. In many cases, an extra undershirt, an extra coat and a blanket will each add comfort and pleasure to the journey.

Always be covered when asleep, for the body is not so warm then as when awake.

Eat heartily. Do not reject a due proportion of fatty foods. Keep the body well nourished.

After speaking, put on an extra coat, and if you are going out into the cold air, protect the throat, and while in the open air use the voice as little as possible. When hoarse, speak as little as possible, else the voice may be permanently injured, or even lost.

Wear overshoes in all damp and sloppy weather. No leather is known which will keep the feet so dry as rubber overshoes. These should not be worn indoors, however, as they make the feet damp and cold by preventing the free exhalation of the perspiration.

Change wet clothing as soon as possible after entering the house. While at work one can wear wet clothing, but never when sitting down or standing still. This precaution is very important. Those who are required to stand in wet and damp places should protect themselves with rubber clothing.

Never go to bed with cold or damp feet. Never take warm drinks freely just before going into the cold, wintry air. Never begin a journey before the breakfast has been eaten. Don't sit at an open car window on a cold morning, or if you are in a heated state.

Never sit on a damp cushion, on the damp ground, or on a cold marble or stone step, if you wish to avoid sore throat or colds.

The best lung protectors are dry feet and comfortable body clothing, no exposure, and no late suppers or dissipation.

The aged and young children have not the same power to resist sudden changes of weather as those in the prime of life; hence, in very cold weather they should be provided with extra clothing and warmth.

To recapitulate: To avoid colds, keep the body warmly clad, clean and in pure air as much as possible, laugh, be cheerful and generous.

### HOME TREATMENT OF COLDS.

Many say, "Do nothing," "It will cure itself," etc. But to cut a cold short several days is worth a good deal, when it can be readily done at the commencement. If the person has full control of his time, there is nothing better than a large bowl of hot ginger tea, to be taken at bedtime, and then the patient to be warmly tucked in bed. Free perspiration will soon start. Any form of hot tea, if taken in abundance, will answer equally well: but if the person is liable to be called up in the night, it will not be safe to try this treatment. A warm bath is also beneficial.

The eliminating organs may properly be excited by unirritating remedies, as liquor ammoniæ acetatis, citrate of ammonia, citrate of potash and chlorate of potash. Dr. L. Beal, of London, recommends the following:

℞ Liquor ammoniæ acetatis........................℥ ij<br>

Spiritus chloroformi........................ʒ ij<br>

Potassæ nitras................................grs. lx<br>

Syrup of squill...............................℥ ss<br>

Aquæ........................................℥ vj.

M. Dose: a tablespoonful in an equal amount of water once in two hours, or less frequently, for three or four days.

Or nearly as good will be:

℞ Liquor ammoniæ acetatis........................℥ ij.

Sig. Dose: a tablespoonful every three hours during the day in a wineglass of water. Or five to ten grains of Dover's Powder may be taken on going to bed.

Purgatives are also beneficial. Many find decided relief from a small dose of Rochelle salts.

The dry treatment has been much praised. In this method, the patient, as nearly as possible, abstains from all fluids or drinks for several days. The cold is said to be shortened, but the cure is nearly as bad as the disease. A more rational plan of treatment is to drink largely of water and warm drinks. This plan is recommended by Dr. Beal.

The opium treatment is used in England. Put into a glass of cold water 20 to 30 drops of laudanum. Sip it slowly for an hour or more, and then retire to bed and cover up warmly.

A drop of the tincture of aconite in a glass of water, taken every hour, is also highly commended.

In infants, slight colds require little treatment more than scupulous cleanliness, warm clothing and bathing the feet with warm mustard water. To promote expectoration, give:

℞ Syrupi ipecacuanha.............................ʒ ij

Spt. æther nitr..................................ʒ j

Syr. simplic.....................................℥ ij.

M. Dose: a teaspoonful every three hours.

Inunction of the nostrils and chest with warm suet generally gives considerable relief to children where thcre is a "tight cold." A very much diluted croton oil applied to the chest is also very beneficial.

### COUGHS.

Coughs differ so much from each other in causation that it is very difficult to give any home prescription.

In an adult, the feet may be soaked in hot mustard water, a mustard plaster applied to the chest, or the chest rubbed with a mixture of sweet oil to which a little croton oil has been added. Or,

℞ Paregoric...........................gtts. xv
Aquæ..................................a wineglassful.
Mix. Take above every three hours. Or,

℞ Ammon. carb............................grt. xv

Syr. seneg...............................ʒ iss

Syr. prun. virgin........................ʒ iss

Syr. tolu................................ʒ ij

Aquæ.....................................fl.-℥ iss.

Mix: Dose: a teaspoonful every two or three hours.

An excellent domestic remedy for a cough is,

℞ Hoarhound leaves.........................℥ j
Water......................................pt. j.
Boil for fifteen minutes; strain out the leaves; add enough sugar to make a nice syrup; boil for fifteen minutes. The dose is a tablespoonful every hour.

It is to be constantly borne in mind that the early stages of many severe diseases, as pneumonia, typhoid fever, smallpox, etc., are ushered in by symptoms which are not readily distinguished from those of an ordinary cold. It is a good rule in all cases to take the best possible care of the body in health, and then, whenever it is deranged, to call in the family physician at once, and implicitly follow his advice. A cold may be a little thing, but it often leads to very serious disorders.

